# Potential of margin reduction for cervical cancer radiotherapy in an online adaptive image-guided workflow

**DOI:** 10.1016/j.phro.2026.100923

**Published:** 2026-02-11

**Authors:** FangHua Li, ShouLiang Ding, ZhanLin Chen, Kai Chen, JunYun Li, XinPing Cao, WeiJun Ye, Yi OuYang, XiaoDan Huang, FoPing Chen

**Affiliations:** Department of Radiation Oncology, State Key Laboratory of Oncology in South China, Guangdong Provincial Clinical Research Center for Cancer, Sun Yat-sen University Cancer Center, Guangzhou 510060, PR China

**Keywords:** Online adaptive radiotherapy, Cervical cancer, Cone-beam CT, Reduced PTV margin, Target volume coverage, OAR sparing

## Abstract

**Background and purpose:**

Adaptive radiotherapy (ART) manages anatomical variations through real-time plan adjustments. Herein, we aimed to evaluate the efficacy of ART in improving target coverage and reducing organ-at-risk (OAR) irradiation doses in cervical cancer.

**Materials and methods:**

A prospective single-arm study enrolled 15 patients with cervical cancer. Participants received definitive chemoradiotherapy (45–50 Gy/25Fractions) using cone-beam computed tomography-guided online ART workflow. Endpoints included geometric miss volume (target volume underdosed), planning target volume (PTV) coverage (V_100%_), OAR sparing (generalized equivalent uniform dose [gEUD] and normal tissue complication probability [NTCP]), and dose homogeneity/conformity parameters (coefficient of variation [CV], Paddick Index [PI]). Mixed-effects models compared ART and simulated image-guided radiotherapy (IGRT) plans, with Wilcoxon signed-rank and Mann-Whitney U tests for statistical analysis.

**Results:**

Among 375 adaptive sessions, ART outperformed IGRT, reduced geometric miss volume by 85.1% (0.28 cm^3^ vs. 1.88 cm^3^; *P* < 0.001) and achieved V_100%_ ≥ 99% in 99.2% of fractions (vs. 75.0% with IGRT; *P* < 0.001). ART improved PTV dose homogeneity (median CV: −0.29% vs. 3.89%; *P* < 0.001) and conformity (median PI: 0.75 vs. 0.60; *P* < 0.001). Additionally, ART reduced rectum gEUD by 4.39% (*P* < 0.001) and NTCP by 52.27% (*P* < 0.001), with similar benefits for other OARs (*P* < 0.001). ART maintained robustness against intrafractional anatomical changes, with minimal target coverage loss and stable OAR doses despite bladder/rectum volume fluctuations. Patients exhibited 100% complete response and mild acute side effects (no grade ≥ 3 enteritis; no cystitis).

**Conclusions:**

Online ART with 3-mm isotropic margins is feasibile for cervical cancer, achieved high target coverage while reduced OAR doses and NTCP.

## Highlights

• Adaptive radiotherapy enabled target volume margin reduction in cervical cancer.
• Adaptive radiotherapy assured target volume coverage while reduced organ irradiation.
• Bladder-filling drinking protocol enhanced small-bowel sparing during radiotherapy.
• Adaptive radiotherapy reduced the normal tissue complication probability of organs.

## Graphical abstract

**Figure:**
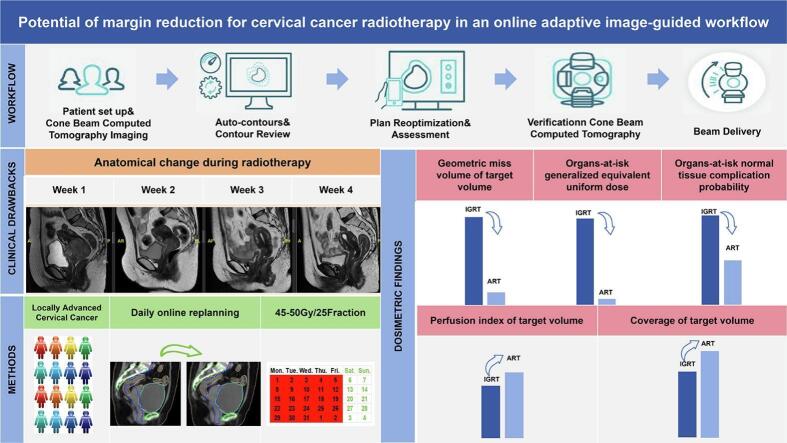

## 1 Introduction

Radiotherapy is a cornerstone treatment for locally advanced cervical cancer (LACC), yet its efficacy is challenged by interfractional and intrafractional anatomical variations. Throughout treatment, uncertainties arise from daily setup errors, organ motion (e.g., bladder and rectal filling), and tumor regression, which may exceed 20 mm in cervical cancer patients [1], [2]. Conventional workflows use internal target volume (ITV) delineation and large planning target volume (PTV) margins (typically 15 ∼ 20 mm) to ensure coverage, but often at the expense of excessive irradiation to organs at risk (OARs) [3], [4]. This “one-size-fits-all” approach inevitably increases acute side effects, with grade ≥ 2 radiation enteritis and cystitis exceeding 30% [5], [6], severely compromising patients' quality of life. Balancing target coverage with OAR protection requires a shift toward adaptive precision radiotherapy.

Recent advances in artificial intelligence (AI)-driven auto-segmentation, real-time treatment planning optimization, and online adaptive radiotherapy (ART) offer promising alternatives [7], [8]. Using daily cone-beam computed tomography (CBCT) to account for anatomical changes, online ART enables dynamic recontouring and margin reduction while maintaining dose coverage. Evidence suggests that PTV margins may be safely reduced to 5 mm with ART without compromising target coverage [9]. However, the conventional ITV and PTV framework—originally designed for image-guided radiotherapy (IGRT)—lacks validation in adaptive settings. Critical questions persist regarding whether reduced PTV margins can preserve adequate tumor control while mitigating treatment-related side effects, and what specific thresholds of intrafractional anatomical motion warrant the implementation of adaptive adjustments. Rigorous validation of novel contouring standards and margin guidelines for ART is therefore essential to address these knowledge gaps.

While prior studies have compared differences in dose-volume parameter between ART and IGRT [10], [11], three critical limitations persist. Firstly, most protocols retain conservative PTV margins, underutilizing ART's potential for OAR sparing [10], [11]. Meanwhile, the impact of intrafraction motion (e.g., bladder emptying) on dose accumulation is poorly understood. Finally, safety analyses often lack clinical endpoint correlation with margin reduction. This prospective study established a CBCT-based online ART protocol with < 30-minute workflow, and evaluated its feasibility through geometric accuracy, therapeutic adaptability, and temporal stability. Our findings seek to provide evidence for margin reduction in ART of cervical cancer.

## 2 Materials and methods

### 2.1 Patient Cohort

This prospective single-arm observational study evaluated the clinical implementation of ART in cervical cancer. Between October 2023 and October 2024, 15 patients with histologically confirmed LACC were enrolled at Sun Yat-sen University Cancer Center (Supplementary material Table S1). All participants underwent standardized pre-treatment evaluations (Supplementary material A) and written informed consent was obtained. The objective response of the patients were evaluated post-treatment at 12-week follow-up according to the RECIST 1.1. criteria [12] and safety data was recorded throughout the study. The study protocol adhered to the principles of the Declaration of Helsinki and was approved by the Institutional Ethics Committee of Sun Yat-sen University Cancer Center (Approval No. XJS2023-031–01).

### 2.2 Adaptive radiotherapy workflow

The ART workflow was implemented on an Elekta Axesse™ linear accelerator (Elekta AB, Sweden) equipped with a HexaPOD™ couch (Elekta AB, Sweden). Pre-treatment daily CBCT scans (120 kVp, 1.5 mAs, 200° rotation) were acquired and rigidly registered to the planning CT using 6 degrees-of-freedom alignment. The CBCT images were converted into high-quality synthetic CT (sCT) using a two-stage deep learning approach: first, CycleGAN-based pixel-to-pixel translation was performed, followed by diffusion model refinement with Denoising Diffusion Probabilistic Models (DDPM) and ControlNet (a constrained diffusion model with additional spatial constraints). An State Administration for Market Regulation approved AI-based auto-segmentation engine (PVMED, Guangzhou, China) preprocessed OAR contours (bladder, rectum, colon, smallbowel, pelvicbone, etc) on the high-quality synthetic CT, while target volumes from the planning CT were propagated via deformable image registration. All auto-contours were reviewed and manually adjusted by certified radiation oncologists (F.P.C., X.D.H., or K.C.).

To account for interfractional anatomical variations, the clinical target volume (CTV) was dynamically adjusted based on daily CBCT-visible soft-tissue boundaries and OAR displacements withour additional ITV margin. A 3-mm isotropic expansion was applied to create the interfractional PTV (iPTV). Electron density (ED) values from the planning CT were mapped to CBCT-derived structures using the “Force ED” function in Monaco v6.2.3 (Elekta AB, Sweden), ensuring accurate Monte Carlo dose calculation (variance < 1%). Plan reoptimization utilized a GPU-accelerated scripting interface with the following priorities: Maintain iPTV coverage (V_100%_ ≥99%); Bowel V_40Gy_ ≤ 30%; Bladder V_45Gy_ ≤ 20%; Rectum V_40Gy_ ≤ 50%. Convergence was achieved within 5 min per case. Following online approval, a second CBCT was acquired pre-delivery to detect intrafractional motion (>1 mm threshold) with corrections applied via HexaPOD™ robotic couch adjustments.

### 2.3 Dose-volume and target coverage evaluation parameter

The primary endpoints evaluated interfractional dose distribution of online ART through four metrics: target underdosage volume (V_under_, cubic centimeter) of the interfractional CTV(iCTV), defined as the volume receiving < 100% of the prescribed dose; coverage of the prescription dose for the iPTV, defined as the volume receiving 100% of the prescription dose (V_100%_); dose homogeneity quantified by coefficient of variation (CV = SD/mean dose); and conformity evaluated with the Paddick Index (PI = TV^2^_PIV_/(TV × PIV), where TV_PIV_ is target volume covered by prescription isodose, TV is target volume, and PIV is total volume of prescription isodose) [13]. Secondary endpoints focused on OAR protection, comprising: dose-volume parameters (e.g., V_45Gy_ defined as percentage volume receiving ≥ 45 Gy); generalized equivalent uniform dose (gEUD); normal tissue complication probability (NTCP) modeled by Lyman-Kutcher-Burman (LKB) formalism [14]. Detailed implementation of gEUD (organ-specific parameters) and NTCP (LKB model) are provided in Supplementary eMethods. These parameters collectively assessed ART's capability to maintain target coverage while minimizing radiation side effect to adjacent organs.

### 2.4 Framework for comparing dose-volume parameters

Our evaluation framework employed a comprehensive dose comparison methodology to systematically assess the dosimetric impact of ART. The analysis was designed to quantify both absolute and relative deviations between adaptive and non-adaptive approaches while accounting for interfractional anatomical variations.

Non-adaptive IGRT plans with a 5-mm PTV margin were generated on planning CTs using institutional protocols, then recalculated on registered CBCT datasets for each fraction using GPU-accelerated Monte Carlo algorithms, serving as the non-adaptive comparator for subsequent analyses. For each fraction (i), we computed the adaptive gain (ΔMetric_i,Structure_) through differential analysis between ART-delivered and IGRT-recalculated doses:

ΔMetric_i,Structure_ = Metric_iART_,_Structure_ − Metric_iIGRT,Structure_

where Metric_iART,Structure_ and Metric_iIGRT,Structure_ represent the value of the dose-volume parameter for the specified structure, as calculated from the plan delivered on fraction i (ART plan) and the original static IGRT plan re-computed on the anatomy of fraction i, respectively. This pairwise comparison allowed direct isolation of adaptation benefits.

To standardize comparisons across patients and structures, we calculated percentage deviations relative to planning CT baselines:

%ΔMetric_i,Structure_ = 100% × (Metric_i,Structure_ − Metric_iplan,Structure_)/Metric_iplan,Structure_

with Metric_iplan,Structure_ representing dose parameters on static planning CT structures.

Recognizing potential intrafractional changes, secondary CBCT pairs (median interval: 21 min) were rigidly registered to the first CBCT image using mutual information algorithm. The ART plans were recalculated on secondary CBCTs to evaluate: target coverage degradation and OAR dose escalation.

### 2.5 Statistical analysis

All statistical computations were conducted using R and SPSS software (Supplementary material A). Non-normally distributed variables are summarized as median [range]. Nonparametric tests included Wilcoxon signed-rank tests for paired comparisons and Mann-Whitney U tests for independent group. A linear mixed-effects model (lme4 package) was used to compare ART and IGRT, with modality as a fixed effect and patient anatomy as a random effect. Dose-volume parameters (e.g., V_under_, PI, gEUD differences) served as dependent variables. Multivariable regression analysis evaluated the impact of interfractional organ volume changes (ΔVolume = daily CBCT-derived volume − planning CT volume) on dose deviations. Robust regression with Huber-White standard errors was applied to mitigate heteroscedasticity, incorporating covariates including baseline OAR volume, fraction number, and patient positioning residuals (6D couch shifts). Collinearity was assessed using variance inflation factors (VIF < 2.0). All tests were two-tailed with significance defined as p < 0.05. Sensitivity analyses included bootstrap resampling (1,000 iterations) for nonparametric confidence intervals [CI] and likelihood ratio tests for mixed-model validity. Effect sizes were reported as β coefficients [95% CI] for regression outcomes and Hodges-Lehmann estimators [CI] for nonparametric comparisons.

## 3 Results

Baseline clinical characteristics and dose-volume parameters are summarized in Table S1. The median age was 56 years (range, 28 to 74), with all patients staged as FIGO 2018 IB-IIIB (n = 10, 66.7%) or IIIC1r-IVA (n = 5, 33.3%). Among 375 adaptive fractions, the online ART workflow required a mean (SD) of 25.2 (5.2) minutes per fraction.

**Fig. 1:**
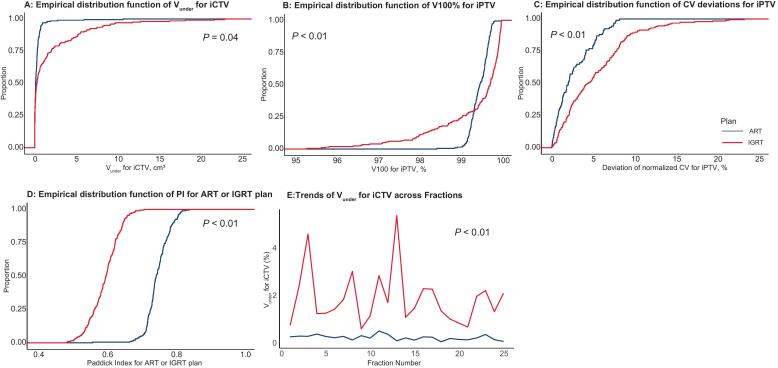
Comparative Dosimetric Parameters Between ART and IGRT for Target Volumes. A. Empirical cumulative distribution functions (ECDFs) of clinical target volume (iCTV) geometric miss (V_under_) for ART and IGRT. B. ECDFs of planning target volume (iPTV) coverage (V_100%_). C. ECDFs of normalized coefficient of variation (CV) for iPTV dose homogeneity. D. ECDFs of iPTV Paddick Conformity Index (PI) between ART and IGRT. E. Longitudinal trends of V_under_ across treatment fractions.

The mean V_under_ of iCTV was 85.1% lower with ART (0.28 ± 0.53 cm^3^) than with IGRT (1.88 ± 3.62 cm^3^), with a median paired difference of 1.61 cm^3^ (95% CI: 1.32 to 1.89 cm^3^) (Fig. 1A). ART also achieved satisfactory iPTV coverage (V100% ≥ 99%) in 99.2% (372/375) of fractions, compared to 75.0% (281/375) with IGRT (p < 0.001). Suboptimal coverage (>1% deviation) occurred in 25.0% of IGRT fractions but was eliminated by ART.

ART plans demonstrated better dose homogeneity (median CV: −0.29% [range, −9.81% to 8.21%] vs. 3.89%[-5.77% to 23.32%]; p < 0.001) and superior conformity (median PI: 0.75[range, 0.56 to 0.84] vs. 0.60 [0.48 to 0.71]; p < 0.001) (Fig. 1). Sensitivity analysis confirmed that ART consistently reduced geometric miss across all fractions (p < 0.001).

After adjusting for interpatient variability and temporal effects, linear Mixed-Effects Models quantified ART’s advantages (Table 1): Geometric miss reduction: ΔV_under_ = -1.64 cm^3^ (95% CI, −2.11 to −1.17; p < 0.001); Coverage improvement: ΔV_100%_ = +0.17% (95% CI, 0.04 to 0.29; p = 0.009); Stability gain: ΔCV % = −5.27% (95% CI, −5.96 to −4.57; p < 0.001); Conformity gain: ΔPI = +0.15 (95% CI, 0.15 to 0.16; p < 0.001). ART's improvements were independent of IGRT results (linear regression slope ≈ 0, r = -0.03; Fig. S1).

**Table 1:** Comparison of dosimetric parameters between adaptive radiotherapy (ART) and image-guided radiotherapy (IGRT) using linear mixed-effects modeling.

**Metric**	**Fixed Effects**	**Fixed-effects coefficients (β)**	**SE**	**95% CI**	**p-value**	**Random Effects Variance (σ^2^)**
**V_under_ of iCTV, cm^3^**	Reference: IGRT	–	–	–	–	σ^2^ = 0.88
	ART vs IGRT	−1.64	0.24	[-2.11, −1.17]	<0.001	SD = 0.94
**V_100%_ of iPTV, %**	Reference: IGRT	–	–	–	–	σ^2^ = 0.07
	ART vs IGRT	0.17	0.06	[0.04, 0.29]	0.01	SD = 0.26
**ΔCV% of iPTV, %**	Reference: IGRT	–	–	–	–	σ^2^ = 5.38
	ART vs IGRT	−5.27	0.35	[-5.96, −4.57]	<0.001	SD = 2.32
**PI of iPTV**	Reference: IGRT	–	–	–	–	σ^2^ = 0.00
	ART vs IGRT	0.15	0.00	[0.15, 0.16]	<0.001	SD = 0.03
**ΔgEUD% of Rectum, %**	Reference: IGRT	–	–	–	–	σ^2^ = 2.11
	ART vs IGRT	−4.1480	0.23	[-4.60, −3.70]	<0.001	SD = 1.45
**ΔgEUD% of Bladder, %**	Reference: IGRT	–	–	–	–	σ^2^ = 0.62
	ART vs IGRT	−7.14	0.27	[-7.67, −6.61]	<0.001	SD = 0.79
**ΔgEUD% of SmallBowel, %**	Reference: IGRT	–	–	–	–	σ^2^ = 17.78
	ART vs IGRT	−6.84	0.57	[-7.97, −5.72]	<0.001	SD = 4.22
**ΔgEUD% of Colon, %**	Reference: IGRT	–	–	–	–	σ^2^ = 64.91
	ART vs IGRT	−6.98	0.52	[-8.00, −5.95]	<0.001	SD = 8.06
**ΔgEUD% of PelvicBone, %**	Reference: IGRT	–	–	–	–	σ^2^ = 0.27
	ART vs IGRT	−7.34	0.16	[-7.65, −7.03]	<0.001	SD = 0.52

Abbreviation: SE = Standard Error; V_under_: geometric miss volume; iCTV: interfractional clinical target volume; iPTV: planning target volume; V_100%_: prescription dose coverage; CV: coefficient of variation; PI: Paddick Index; gEUD: generalized equivalent uniform dose.

Online ART demonstrated superior OAR sparing compared to IGRT, particularly for rectum, bladder,

and pelvic bone protection. As illustrated in Fig. S2, ART plans systematically reduced high-dose exposure, with mean percentage deviation in normalized rectum gEUD was −4.39% (SD: 1.76%) for ART versus −0.24% (SD: 3.33%) for IGRT (p < 0.001). This improvement translated into a clinically meaningful reduction in side effect risk: the normalized NTCP for the rectum decreased by −52.27% (SD: 15.56%) with ART but increased by 10.35% (SD: 59.4%) with IGRT (p < 0.001). Similar benefits were observed for the bladder, small bowel, colon, and pelvic bone, with ART achieving both gEUD and NTCP reductions (Fig. S3). Conventional dose metrics (e.g., D_2cc_, V_45Gy_, V_40Gy_) also confirmed significant reductions in high-dose volumes and irradiated OAR regions (p < 0.001). Scatterplots of normalized gEUD deviations (Fig. S4) showed no correlation between ART and IGRT plans relative to baseline for the rectum, bladder, or pelvic bone (slope ≈ 0, |r| < 0.3), indicating that ART adaptively counteracts overdosing, while IGRT is more affected by anatomical variations.

**Fig. 2:**
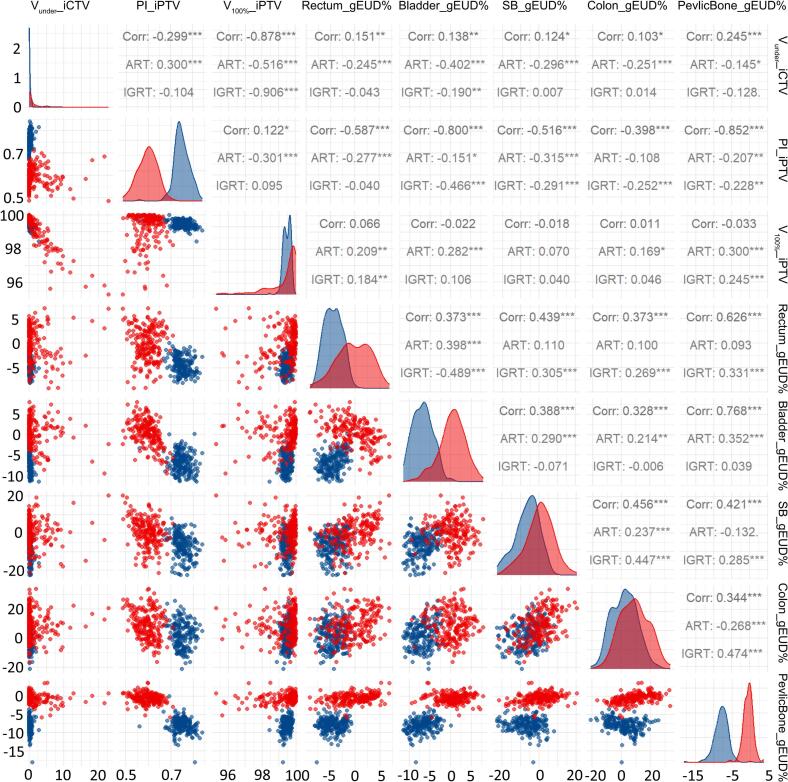
Multivariate Dosimetric Correlations in ART and IGRT. Faceted scatter matrix comparing dose-volume parameters (target coverage, OAR sparing, homogeneity). Diagonal: Histograms of parameter distributions. Off-diagonal: Pairwise correlations (Pearson’s *r*). Significance levels: ***p < 0.001, **p < 0.01, *p < 0.05. Red and blue points represent IGRT and ART parameters, respectively, with distinct clustering demonstrating ART’s improved control over OAR doses while maintaining target coverage. (For interpretation of the references to colour in this figure legend, the reader is referred to the web version of this article.)

Direct per-fraction comparisons (Fig. S5) showed that ART significantly reduced gEUD while maintaining target coverage: rectum by 4.15 Gy (SD: 3.41), bladder by 7.14 Gy (4.01), small bowel by 6.84 Gy (3.24), colon by 6.98 Gy (2.69), and pelvic bone by 7.33 Gy (2.60). Mixed-effects models confirmed that OAR sparing was independent of target coverage improvements (p < 0.01) as shown in Table 1. A strong negative correlation between V_under_ and V_100%_ (r =  − 0.878, p < 0.001) indicates that larger CTV underdosing volumes compromise PTV coverage (Fig. 2). However, ART demonstrated weaker correlation (r =  − 0.516) than IGRT (r =  − 0.906), indicated greater robustness to underdosing. Volcano plots (Fig. S6) further confirmed ART’s capacity to reduce iCTV underdosing (negative mean difference in V_under_) while enhancing PTV coverage (positive trend in V_100%_) and reducing NTCP and high-dose volumes to pelvic OARs.

**Fig. 3:**
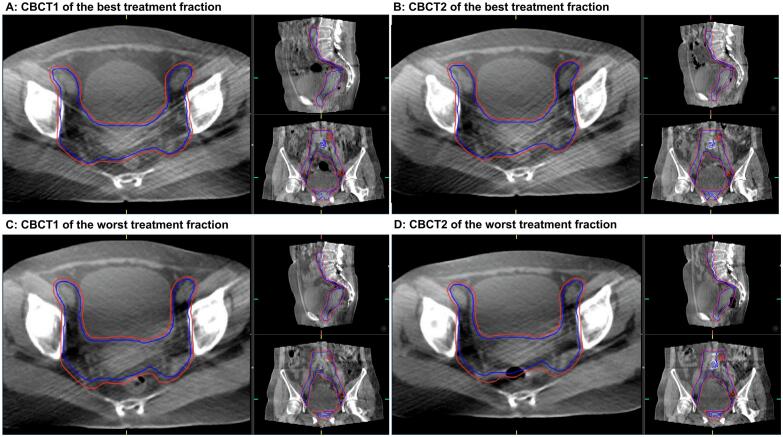
Representative images of adaptive CTV and PTV contours on CBCT pairs of one patient The best treatment fraction (A-B) and the worst fraction (C-D) of one patient were shown. The adaptive CTV(bule) and the 3-mm PTV (red) contours were superimposed after pre- and posttreatment CBCT matching with respect to bony anatomy. During the best treatment fraction (A-B), 100% coverage with minimal anatomical variations were observed on the two set of CBCTs over a 20-minute intrafraction interval. Even during the worst treatment fraction when the bowel movement on the CBCT2 (D) was rather obvious compared to that on the CBCT1 (C), still the adaptive CTV and 3-mm PTV margin ensures the target coverage on the CBCT2. (For interpretation of the references to colour in this figure legend, the reader is referred to the web version of this article.)

**Fig. 4:**
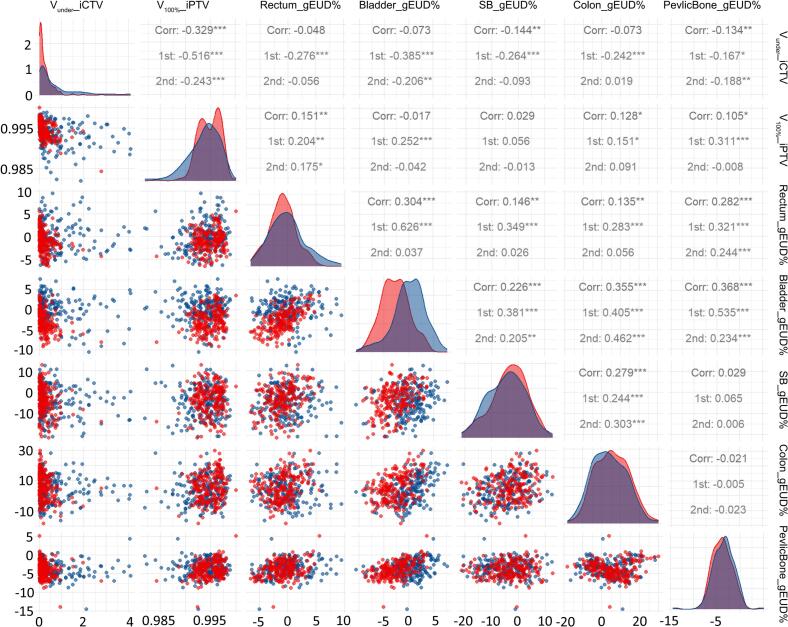
Validation of Dosimetric Robustness for ART. This faceted scatterplot matrix evaluates the dosimetric robustness of intrafractional ART plans. Red points represent the initial ART plan (1st), while blue points denote the secondary plan (2nd) generated by recalculating the initial ART plan on the second pre-treatment CBCT dataset. Each subplot illustrates correlations between distinct dosimetric parameters, with correlation coefficients (Corr) and significance levels (***p < 0.001, **p < 0.01, *p < 0.05) indicating inter-group differences. (For interpretation of the references to colour in this figure legend, the reader is referred to the web version of this article.)

The ART plans maintained robust dosimetric performance over a 20-minute intrafraction interval (Fig. 3). Compared to the initial plan, the second plan exhibited marginally increase in iCTV geographic miss volume (median 0.08 cm^3^, range: −1.40 to 4.28 cm^3^) (Fig. 4). OAR doses were comparable to the initial plan, except for the bladder, where dose escalation correlated with volumetric expansion.

**Fig. 5:**
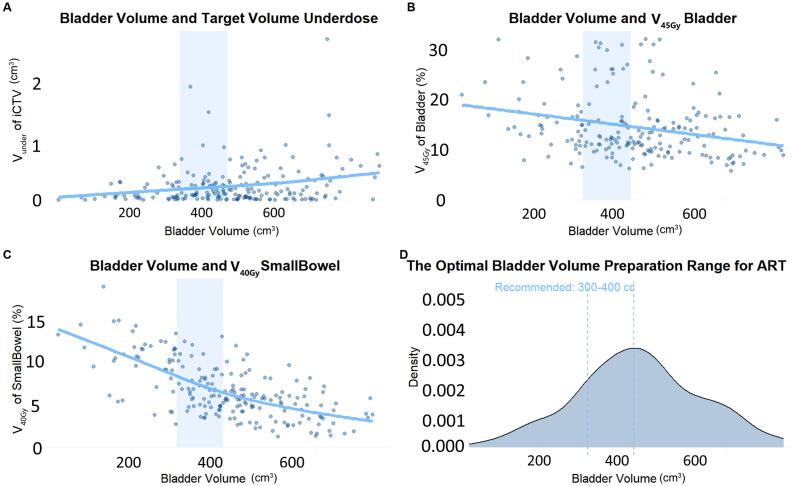
Impact of Bladder Volume on Target Missed Volume and Bladder/Small Bowel Dosimetry in ART Workflow. A. Interfractional Clinical Target Volume (iCTV) Missed Volume (V_under_) and Bladder Volume. B. Bladder Volume and Bladder V_45Gy_ Correlation. C.Linear reduction in small bowel V_40Gy_ with bladder filling. D. Density Distribution of Bladder Volumes with Recommended Range.

Under IGRT, bladder/rectum volume changes were associated with target misses and OAR dose variations (Table S2), though consistent bladder filling to 300 to 400 cm^3^ mitigated these risks (Fig. S7). For ART, adaptive replanning reduced sensitivity to bladder volume changes, but maintaining bladder filling at 300 to 400 cm^3^ still improved small bowel sparing (Fig. 5). Analysis of intrafractional organ volume dynamics revealed a mean bladder volume increase of 90.38 cm^3^ (SD: 61.39) and a minor rectum volume increase of 1.67 cm^3^ (SD: 9.64) during ART sessions. Stable volumes helped maintain coverage, but significant bladder distension or rectal reduction increased geographic miss and reduced coverage.

At 12-week follow-up, all patients (100%) achieved complete response based on imaging and clinical evaluation. Radiation-induced enteritis occurred in 5 patients (33.3%) with grade 1 symptoms (mild diarrhea or transient abdominal discomfort) and 2 patient (13.3%) with grade 2 enteritis (moderate diarrhea requiring symptomatic management). No grade ≥ 3 enteritis (e.g., obstruction, perforation, or hemorrhage) or any cases of cystitis (acute or chronic) were reported.

## 4 Discussion

This prospective study validated the clinical feasibility of a streamlined online ART framework for cervical cancer, which adopted a 3-mm isotropic PTV margin. By enabling daily adaptive adjustments, this strategy not only minimizeed geometric target miss and significantly spared OARs relative to conventional IGRT but also maintained robust performance against interfractional anatomical variations, thereby resolving long-standing debates regarding the safety and practicality of margin reduction in pelvic ART. Beyond these immediate dosimetric benefits, the framework represented a paradigm shift in radiotherapy planning: it leveraged daily recontouring to redefine the role of PTV margins, moving away from a primary focus on capturing anatomical motion toward compensating for residual systematic uncertainties—including those inherent to CBCT image resolution limits. This shift underscored ART’s transformative potential to advance precision radiotherapy for cervical cancer, balancing stringent target coverage with maximal normal tissue protection.

The observed 85.1% reduction in geometric miss volume (V_under_) with ART corroborates emerging evidence that margin reduction below 5 mm becomes viable when coupled with daily soft-tissue adaptation. While Van Herk’s formula [15], [16] recommended 7.5-mm margins for this population, our 3-mm protocol achieved uncompromised CTV coverage (V_100%_ ≥99% in 99.2% of fractions), suggesting that population-based internal margins overestimate residual uncertainty in adaptive workflows. Dual-path dose accumulation revealed that conventional ITV/PTV approaches [17], [18] overestimated OAR doses by 32.7% (rectum V_45Gy_) to 41.9% (small bowel V_40Gy_), indicating that real-time adaptation makes static motion buffers unnecessary. The safety of reduced margins was validated through three-dimensional robustness analysis. Our data showed that 3-mm margins remained effective even under intrafractional bladder distension, demonstrating ART’s ability to proactively manage overdose risks—a key advantage over IGRT’s reactive adjustments [19], [20].

The inverse correlation between consistent target coverage (V_100%_) and OAR dose variability (r =  − 0.516 for ART vs. − 0.906 for IGRT) highlighted a key advantage of adaptive workflows: Daily ART effectively neutralised the impact of organ motion, so OAR sparing depended more on the quality of the initial plan than on unpredictable anatomical shifts. This represents a paradigm shift—where IGRT forced a trade-off between target and OAR doses, ART transformed OAR dose variability into a function of planning quality [21], [22], [23]. Specifically, the linear relationship confirmed that adaptive workflows amplify the impact of initial optimization, effectively preserving the intended therapeutic ratio [24]. This explains why ART maintained stable OAR doses despite uterine motion, while IGRT showed cumulative deviations. Such robustness allows ART to transcend traditional motion-compensation methods, converting variable anatomy into predictable dose delivery.

The advantages of ART on dose distribution translated into clinically meaningful side effect reductions. NTCP modeling predicted significant risk reduction for grade ≥ 2 radiation proctitis and radiocystitis without dose escalation. This validateed that adaptation—without dose escalation or hypofractionation—can achieve side effect profiles competitive with advanced modalities. Crucially, the synergy between margin reduction and AI-driven auto-planning enabled workflow feasibility: 99.5% of cases were reoptimized within 5-minute. This efficiency gain, coupled with GPU-accelerated workflows (25.2 min/fraction), addressed a key barrier to clinical adoption, positioning ART as a viable alternative to conventional IGRT for curative intent treatments.

Despite the inherent adaptability of ART to bladder volume fluctuations, our data revealed an intricate trade-off between bladder filling stability and small bowel sparing. Quantitative analysis identified a J-curve relationship: optimal bladder volumes between 300 to 400 mL (SD < 20%) reduced small bowel V_40Gy_ compared to irregular filling states, underscoring the necessity of patient education and protocolized bladder preparation even within adaptive workflows. Therefore, we propose triggering mandatory reoptimization based on bladder volumetry: eg. triggering reoptimization when bladder volumes deviations > 30% [25]. Importantly, suboptimal bladder filling in IGRT correlated with geometric miss [26], [27], whereas ART mitigated such risks despite variations, demonstrating the complementary roles of protocol adherence and adaptive technology. Future integrations of AI predictive models, trained on daily CBCT time-series data, could further personalize bladder preparation thresholds based on individual anatomical elasticity and intrafractional voiding patterns.

While this study provided evidence for ART’s superiority on dose distribution, its single-arm design warrants validation; thus we have initiated a randomized controlled trial (ChiCTR2400088678) to further validate both efficacy and side effect outcomes with long-term follow-up [28]. Additionally, the trade-off between prolonged ART workflows (50% longer than IGRT) and side effect reduction merits further cost-effectiveness analysis. Future integration of AI-based motion prediction—using initial CBCT to model individual displacement trajectories—could enable trigger-based ART, reserving daily replanning for anatomically unstable patients.

In conclusion, by transcending the rigid ITV/PTV paradigm, this study established online ART as a transformative strategy in cervical cancer radiotherapy. The 3-mm margin protocol achieved motion-agnostic dose distribution, turning anatomical uncertainty into a manageable variable. Supported by evidence of improved therapeutic precision and reduced side effects, ART contributed to advancing benchmarks in pelvic radiotherapy and suggested a potential pathway toward more personalized adaptation.

## 5 Declarations

Ethics approval and consent to participate this study was conducted in accordance with the ethical standards of Institutional Review Board (IRB) of Sun Yat-sen University Cancer Center (Approval No. XJS2023-031-01).

## 6 Availability of data and materials

Not applicable.

## Funding

This study was supported by grants from the China Primary Health Care Foundation (chmdf2025-xrky02-19) and Guangzhou Health and Wellness Science and Technology Project (20231A011120).

## Declaration of competing interest

The authors declare that they have no known competing financial interests or personal relationships that could have appeared to influence the work reported in this paper.
