# Supplementary material for: Potential of margin reduction for cervical cancer radiotherapy in an online adaptive image-guided workflow
Source: Phys Imaging Radiat Oncol. 2026 Feb 11;37:100923. doi: 10.1016/j.phro.2026.100923 (PMC12925179; doi:10.1016/j.phro.2026.100923)

eMethods.

Patients with histologically confirmed locally advanced cervical cancer were referred to the radiation oncology department. Eligible patients met the following inclusion criteria: histologically confirmed locally advanced cervical squamous cell carcinoma, adenocarcinoma, or adenosquamous carcinoma; scheduled for definitive chemoradiotherapy (concurrent platinum-based chemotherapy); no prior radical hysterectomy or pelvic radiotherapy; Eastern Cooperative Oncology Group performance status ≤2. Exclusion criteria included: history of other malignancies within 5 years (except papillary thyroid carcinoma or basal cell skin cancer); previous radiotherapy to any body region; patient refusal to participate; severe comorbidities precluding treatment completion (e.g., uncontrolled inflammatory bowel disease); documented psychiatric disorders impairing protocol compliance. All participants underwent standardized pretreatment evaluations comprising: clinical assessment (pelvic examination, ECOG score); contrast-enhanced pelvic MRI and PET/CT for staging; hematologic and biochemical profiling (complete blood count, renal/liver function). Two independent radiation oncologists (F.P.C. and X.D.H.) reviewed imaging and assigned FIGO 2018 stages. After that, patients were scheduled to undergo definitive radiotherapy.

Treatment Planning Simulation and Imaging

All patients underwent a simulation session for treatment planning, which included both contrast-enhanced and non-contrast-enhanced planning CT scans. Imaging was performed with the patient positioned supine on a vacuum bag immobilization device to ensure reproducibility. Patients were instructed to follow specific preparation protocols, including bladder filling and emptying, as well as bowel preparation, to minimize organ motion and inter-fractional variability. Reference plans were generated using the Monaco treatment planning system (Elekta), adhering to institutional protocols for adaptive radiotherapy (ART).

Target Volume Delineation and Contouring

Target volumes were defined based on pre-radiotherapy MRI and CT imaging (PreRT-MR/CT) in conjunction with gynecological examination. The gross tumor volume of lymph nodes (GTVnd) and clinical target volume (CTV) were contoured according to the adaptive radiotherapy contouring protocol. The planning target volume (PTV) was generated by expanding the CTV by a uniform margin of 3 mm to account for setup errors and intrafractional motion. Organs at risk

(OARs), including the bladder, rectum, small bowel, and pelvic bones, were meticulously contoured on the planning CT to ensure dose constraints were met and to prevent hotspots in radiosensitive regions.

Adaptive Radiotherapy Workflow

ART was implemented using the Axesse™ accelerator (Elekta), which supports both image-guided radiotherapy (IGRT) and ART modes within an established adaptive workflow. Daily IGRT was performed as a standard component of each radiotherapy session to assess anatomical changes. Online matching was achieved using a 6-degree-of-freedom treatment couch to align the patient's anatomy with the reference plan. Rigid registration was employed to update the isocenter position, ensuring accurate delivery of radiation doses.

Generalized Equivalent Uniform Dose (gEUD) and Normal Tissue Complication Probability (NTCP) Modeling

The gEUD and NTCP of OARs were calculated using the Lyman-Kutcher-Burman (LKB) model [1-4], with organ-specific parameters derived from prior clinical studies:

Organ	TD50 (Gy)	M	N
Bladder	80	0.15	0.15
Rectum	76.9	0.13	0.09
SmallBowel	55	0.12	0.15
Colon	65	0.11	0.15
PelvicBone	32	0.175	1

To facilitate inter-patient comparisons, all gEUD and NTCP values were normalized to the prescription dose (e.g., 45~50 Gy for pelvic fields) using a standardized scaling protocol. A team of experienced medical physicists and professional radiation oncologists analyzed all adaptive plans, deformed images, and structures. The medical physicists were responsible for the technical aspects of dose calculation, plan optimization, and verification. They used specialized software to perform dose - volume histogram (DVH) analysis and ensure the accuracy of dose calculations. The radiation oncologists focused on the clinical relevance of the plans, including target coverage and OAR sparing. They reviewed the contouring of structures to ensure that they were in accordance with the clinical guidelines. Any discrepancies between the physicists and oncologists

were resolved through discussion and consensus.

Statistical software

All statistical computations were conducted using R software (version 4.4.1; R Foundation for Statistical Computing) and IBM SPSS Statistics (version 27.0, IBM Corp).

References.

1. Michalski JM, Gay H, Jackson A, Tucker SL, Deasy JO. Radiation Dose-Volume Effects in Radiation-Induced Rectal Injury. Int J Radiat Oncol Biol Phys. 2010;76:S123–S129.

2. Thor M., Olsson C., Oh J.H., Petersen S.E., Alsadius D., Bentzen L., et al. Urinary bladder dose-response relationships for patient-reported genitourinary morbidity domains following prostate cancer radiotherapy. Radiother Oncol. 2016;119(1):117–122.

3. Fiorino C., Valdagni R., Rancati T., Sanguineti G. Dose-volume effects for normal tissues in external radiotherapy: pelvis. Radiother Oncol. 2009;93(2):153–167.

4. Jose G Bazan, Gary Luxton, Edward C Mok, Albert C Koong, Daniel T Chang. Normal tissue complication probability modeling of acute hematologic toxicity in patients treated with intensity-modulated radiation therapy for squamous cell carcinoma of the anal canal. Int J Radiat Oncol Biol Phys. 2012 Nov 1;84(3):700-6.

Table S1. Patient demographics and clinical characteristics (N = 15).

Characteristics	Values
Age, years	
Median	56
Range	28-74
Histology	
Squamous cell carcinoma	14 (93.3%)
Adenocarcinoma	1 (6.7%)
FIGO stage	
IB-IIIB	10 (66.7%)
IIIC1r-IVA	5 (33.3%)
Baseline PTV volume, cc	
Median	754.4
Range	639.4-841.1
Prescription	
Prescription Dose, Gy	45-50
Fraction	25
Baseline $V_{100\%}$ of PTV, %	
Median	100
Range	99.9-100
Baseline V_{45Gy} of Bladder, %	
Median	20.9
Range	17.8-36.9
Baseline V_{45Gy} of Rectum, %	
Median	54.6
Range	30.2-63.2
Baseline V_{40Gy} of SmallBowel, %	
Median	10.0
Range	6.3-12.9
Baseline V_{40Gy} of Colon, %	
Median	8.0
Range	4.9-13.9
Baseline V_{20Gy} of PelvicBone, %	
Median	70.1
Range	67.0-80.1
Complete response rates	
IB-IIIB	100%
IIIC1r-IVA	100%

Table S2. Multivariable Regression Analysis of Interfractional Bladder/Rectum Volume Changes on the Dosimetric Metrics of Targets and Organs in IGRT approach.

Variable	β Coefficient (95% CI)	p-value	Adjusted R^2	VIF
**V_{under} of iCTV**				
$\Delta V_{Bladder}$, cm^3	-0.01 [-0.02, -0.00]	0.01	0.03	1.07
ΔV_{Rectum}, cm^3	-0.02 [-0.08, 0.04]	0.59		1.07
**gEUD% of Rectum**				
$\Delta V_{Bladder}$, cm^3	-0.01 [-0.01, -0.00]	<0.01	0.23	1.07
ΔV_{Rectum}, cm^3	0.11 [0.06, 0.16]	<0.01		1.07
**gEUD% of Bladder**				
$\Delta V_{Bladder}$, cm^3	0.01 [0.01, 0.01]	<0.01	0.27	1.07
ΔV_{Rectum}, cm^3	-0.04 [-0.08, 0.00]	0.08		1.07
**gEUD% of SmallBowel**				
$\Delta V_{Bladder}$, cm^3	-0.03 [-0.04, -0.02]	<0.01	0.53	1.07
ΔV_{Rectum}, cm^3	0.10 [0.02, 0.19]	0.02		1.07
**gEUD% of Colon**				
$\Delta V_{Bladder}$, cm^3	-0.02 [-0.03, -0.01]	<0.01	0.16	1.07
ΔV_{Rectum}, cm^3	0.28 [0.13, 0.43]	<0.01		1.07
**gEUD% of PelvicBone**				
$\Delta V_{Bladder}$, cm^3	-0.00 [-0.00, -0.00]	0.05	0.02	1.07
ΔV_{Rectum}, cm^3	0.01 [-0.01, 0.03]	0.042		1.07

VIF: variance inflation factor. $\Delta V_{Bladder}$/ΔV_{Rectum} denote the differences between the fractional bladder/rectum volume and the baseline bladder/rectum volume from planning CT

Figure legends

Figure S1. Correlation Analysis of Target Dosimetric Parameters Between ART and IGRT plans.

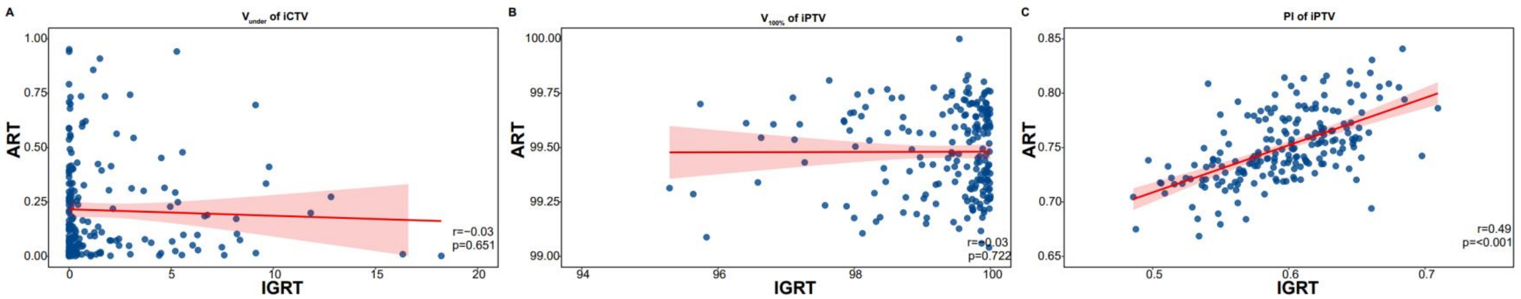

Figure S2. Comparison of gEUD Deviations and NTCP Deviations Between ART and IGRT Plans for OARs.

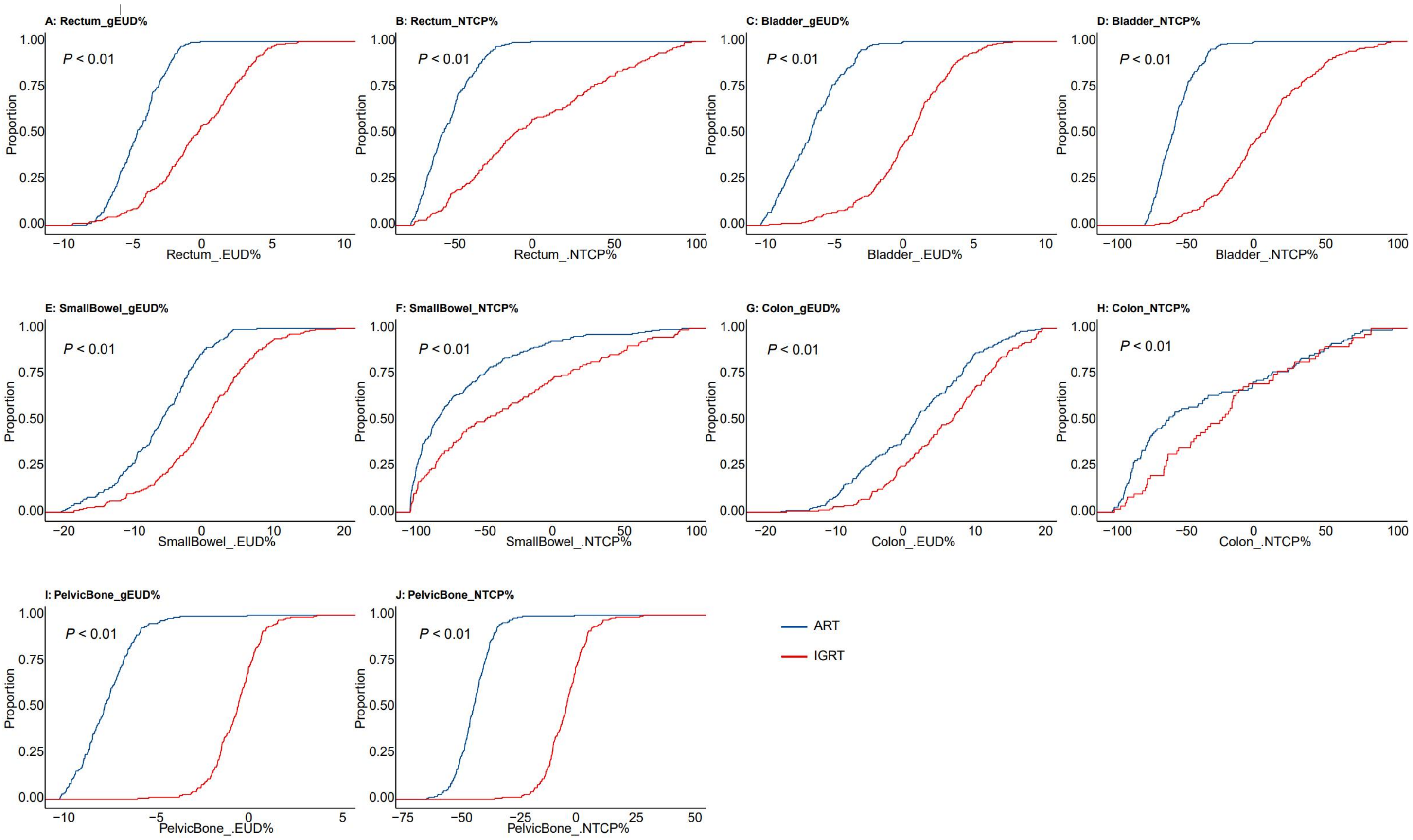

Figure S3. Comparative Distribution of Conventional OAR Dose Metrics Between ART and IGRT plans.

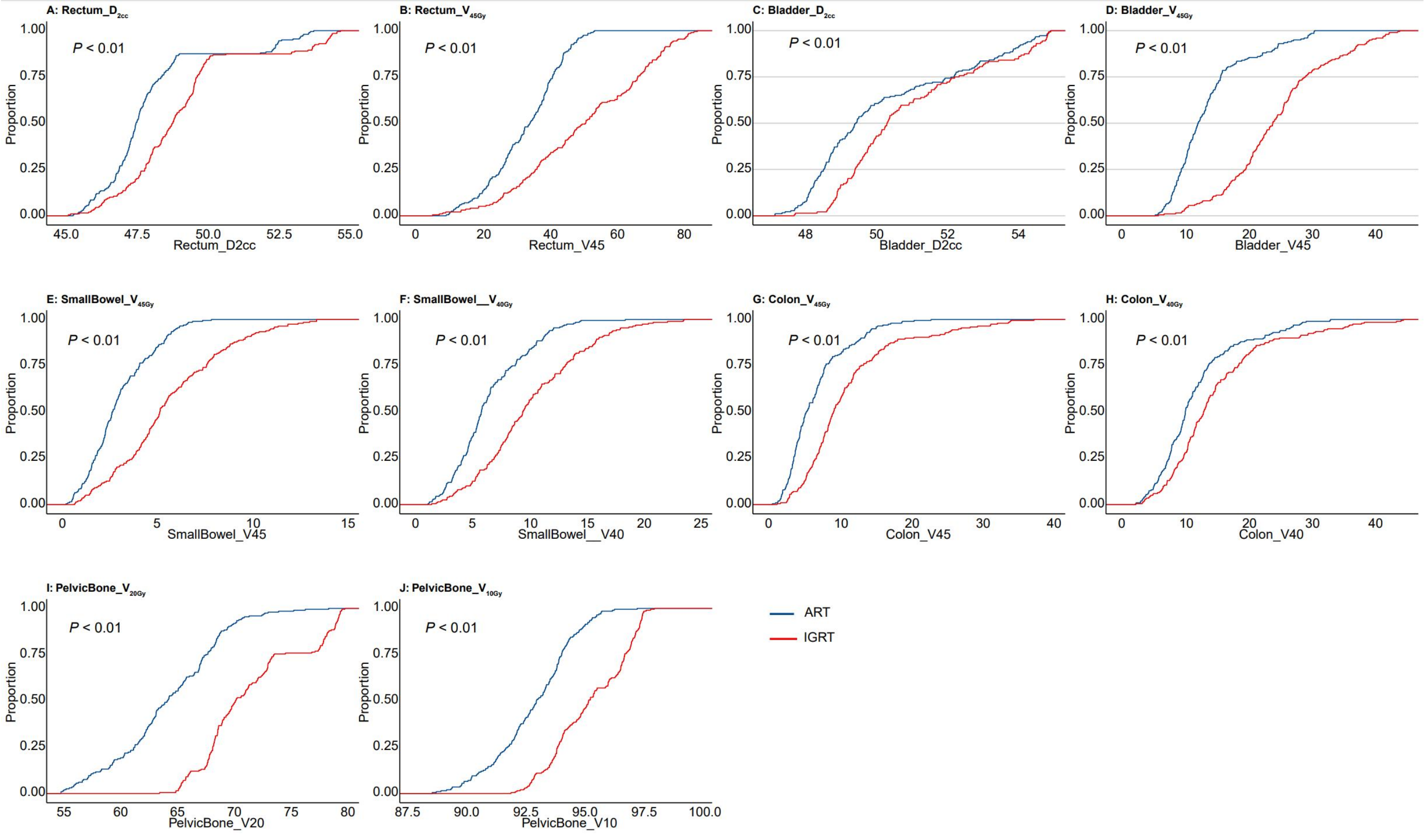

Figure S4. Correlation of OAR gEUD Deviations Between ART and IGRT plans.

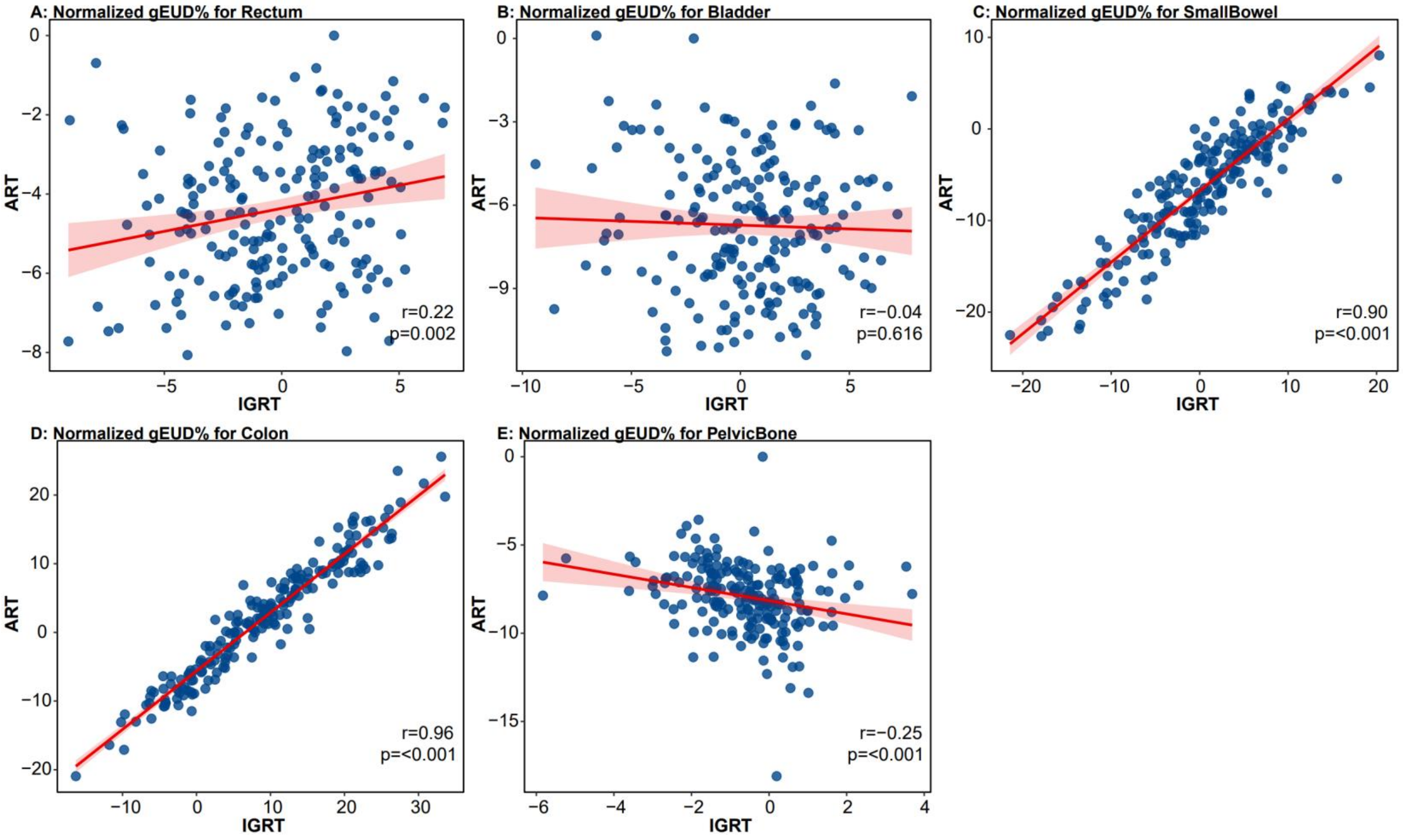

Figure S5. Correlation Between Target Coverage Improvements and OAR Sparing with ART or IGRT.

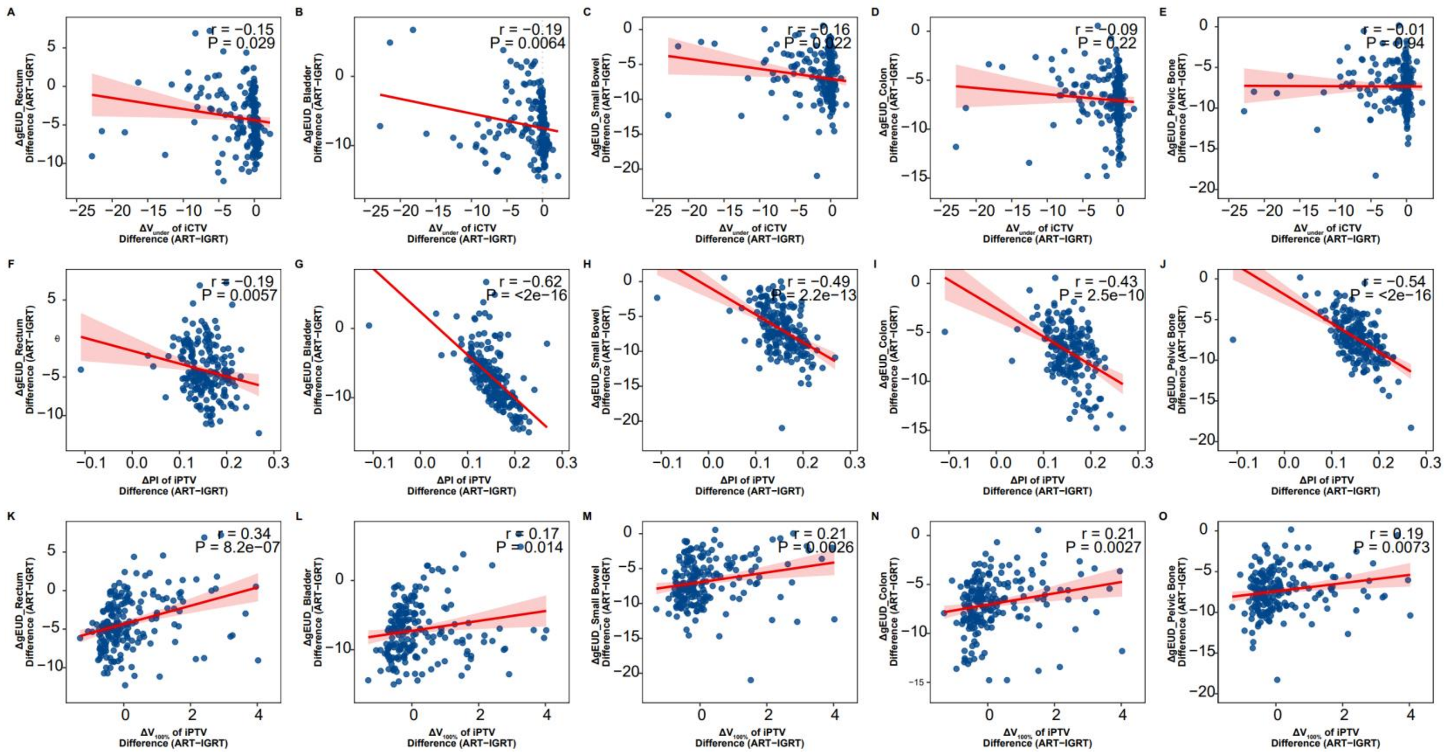

Figure S6. Volcano Plot of Dosimetric Advantages in ART over IGRT across Critical Parameters.

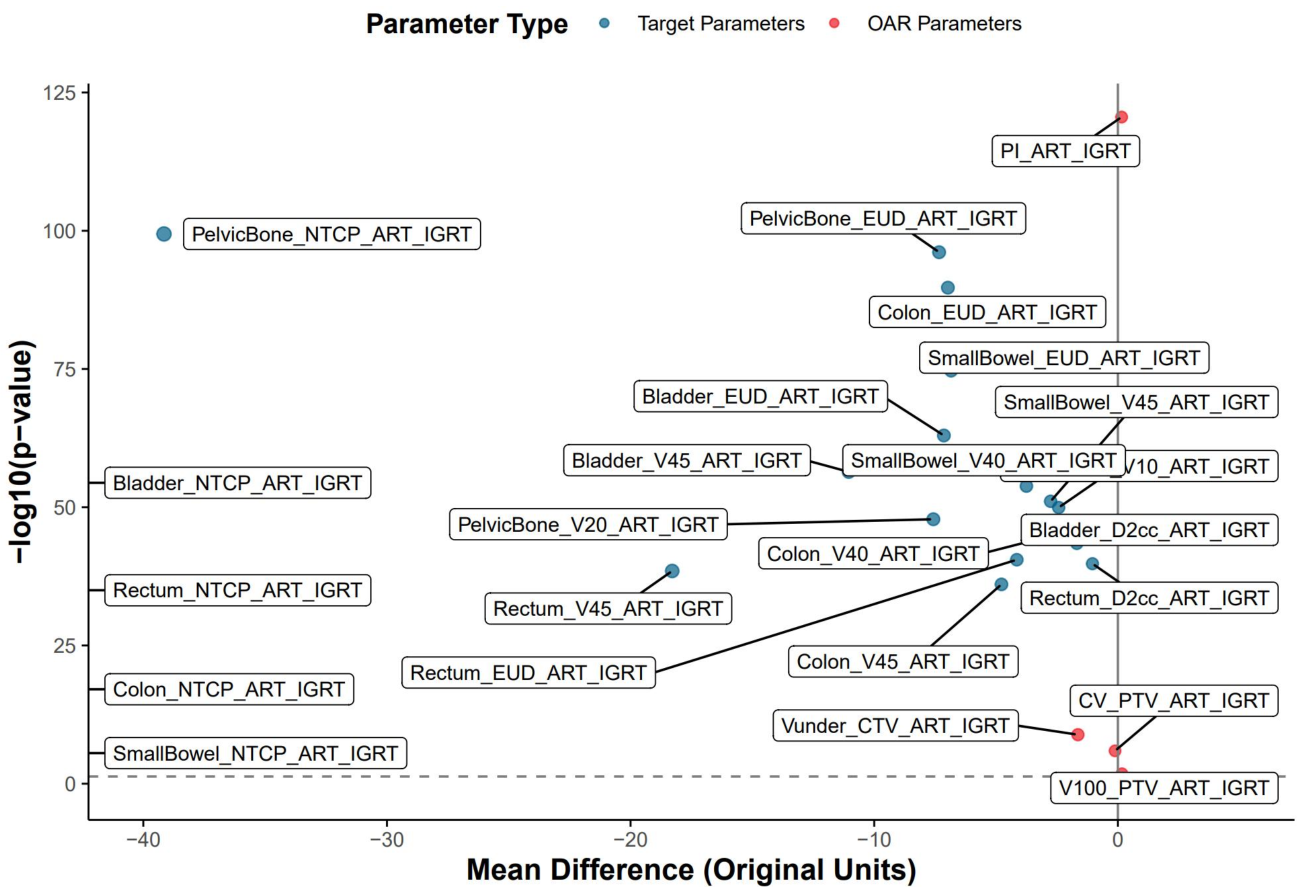

Figure S7. Impact of Bladder Volume on Target Missed Volume and Bladder/Small Bowel Dosimetry in IGRT Workflow.

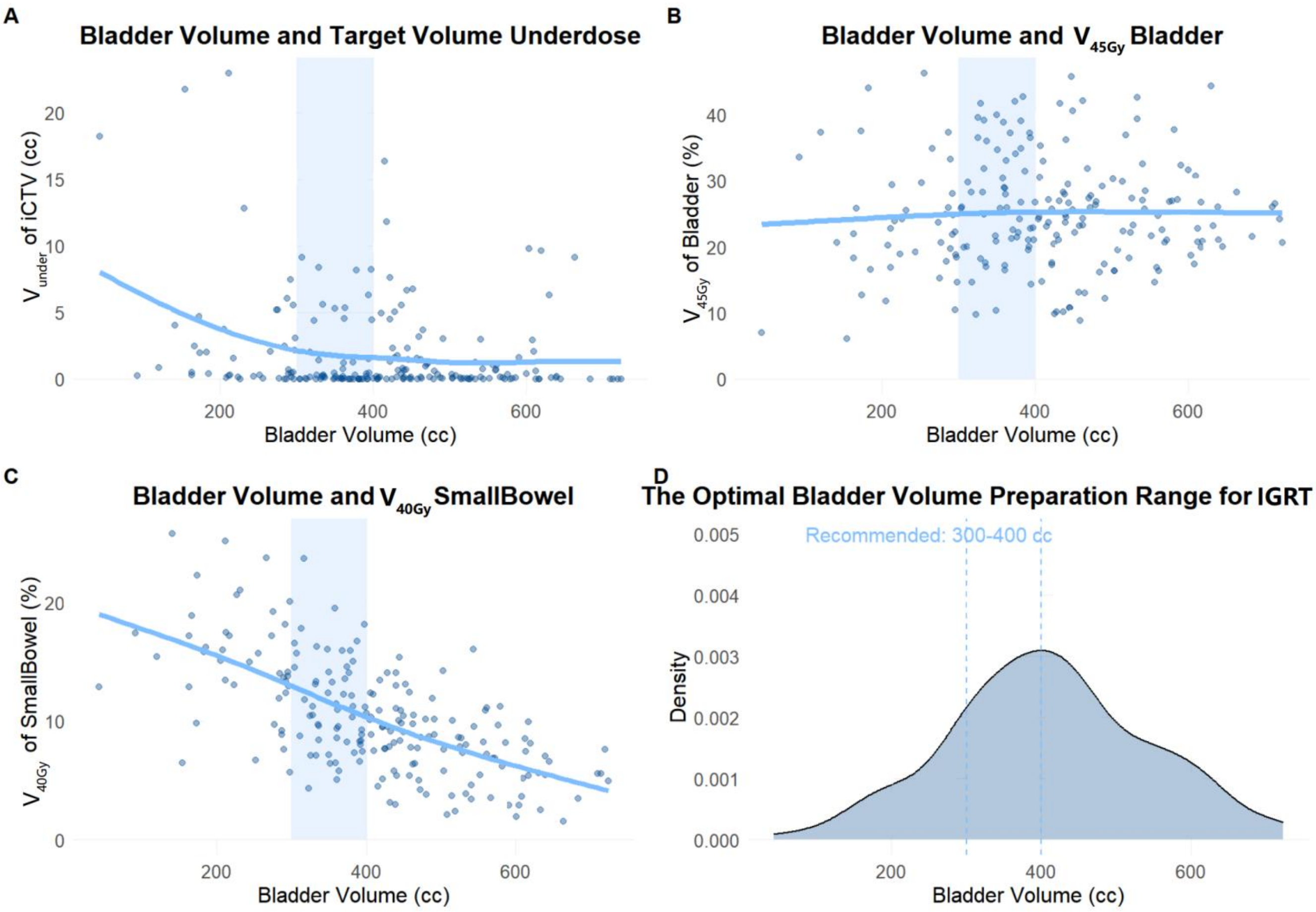

Supplement: Supplementary Data 1 [file mmc1.pdf]
